# Elevated Levels of Serum Thymidine Kinase 1 Predict Poor Survival for Patients with Metastatic Prostate Cancer

**DOI:** 10.1016/j.euros.2024.10.010

**Published:** 2024-10-25

**Authors:** Teemu J. Murtola, Aino Siltari, Paavo Raittinen, Teuvo L.J. Tammela, Stig Linder, Anita Csizmarik, Gero Kramer, Tibor Szarvas

**Affiliations:** a Faculty of Medicine and Health Technology, Tampere University, Tampere, Finland; b Department of Urology, TAYS Cancer Center, Tampere, Finland; c Department of Pharmacology, University of Helsinki, Helsinki, Finland; d Department of Mathematics and Systems Analysis, Aalto University, Helsinki, Finland; e Biomedical and Clinical Sciences, Linköping University, Linköping, Sweden; f Department of Oncology-Pathology, Karolinska Institute, Stockholm, Sweden; g Department of Urology, Semmelweis University, Budapest, Hungary; h Department of Urology, Medical University of Vienna, Vienna, Austria; i Department of Urology, University of Duisburg-Essen, Essen, Germany

**Keywords:** Prostate cancer, Thymidine kinase 1, Survival, Treatment prediction, Antiandrogen, Docetaxel

## Abstract

**Background and objective:**

Prostate-specific antigen (PSA) is of limited value as a surrogate marker for overall survival (OS) in prostate cancer (PC). Serum thymidine kinase 1 (sTK1) is an enzyme expressed by actively dividing cells. Our aim was to evaluate the value of sTK1 as prognostic biomarker in metastatic hormone-sensitive PC (mHSPC) and metastatic castration-resistant PC (mCRPC).

**Methods:**

sTK1 was examined in three cohorts: (1) 43 men with de novo mHSPC managed with androgen deprivation monotherapy; (2) 99 patients with mCRPC managed with androgen receptor signaling inhibitors (ARSIs); and (3) 98 patients with mCRPC treated with docetaxel. sTK1 levels were determined at treatment initiation. OS was evaluated using Cox regression analysis.

**Key findings and limitations:**

In the mHSPC cohort, sTK1 levels in the highest quartile were associated with OS (hazard ratio [HR] 7.77, 95% confidence interval [CI] 2.25–26.9) in comparison to the lowest quartile after multivariable adjustment for age, Gleason score, and PSA. Similarly, sTKI was associated with poor OS in the mCRPC group treated with ARSIs (upper quartile: HR 5.22, 95% CI 2.23–12.2) after multivariable adjustment for age, PSA, and Eastern Cooperative Oncology Group performance status. In the docetaxel-treated mCRPC group the association between OS and sTK1 was lower but still significant (multivariable-adjusted HR 2.28, 95% CI 1.13–4.60). Limitations include the nonrandomized inclusion of patients for different treatments, which could lead to selection bias.

**Conclusions and clinical implications:**

sTK1 predicted OS in mHSPC and mCRPC, demonstrating additional value over established clinical risk factors. sTK1 should be measured in randomized clinical trials of treatments for advanced PC to validate its predictive value.

**Patient summary:**

We found that for patients with metastatic prostate cancer, high levels of a protein called TK1 that is involved in cell division was linked to higher risk of death. Our findings need to be confirmed in other studies.

## Take Home Message

Serum thymidine kinase 1 (sTK1) could be used as a biomarker for risk stratification of patients with metastatic hormone-sensitive or castration-resistant prostate cancers. High sTK1 levels could be used to identify patients at high risk and for tailoring treatment according to disease prognosis.

## 1 Introduction

Prostate cancer (PC) is the most common noncutaneous malignancy among men in developed countries and the second or third most common cause of cancer-related death [1]. The cornerstone for management of metastatic hormone-sensitive PC (mHSPC) is androgen deprivation therapy (ADT) combined with taxane-based chemotherapy (docetaxel or cabizataxel) or androgen receptor signaling inhibitors (ARSIs) such as enzalutamide and abiraterone [2], [3], [4]. mHSPC frequently progresses to metastatic castration-resistant PC (mCRPC) despite ADT and combination treatments. Well-established treatment options for mCRPC include ARSIs, taxanes, and radium-223 [5]. Recently, triplet therapy where ADT is combined with ARSI and docetaxel has emerged as a valuable treatment option for patients with mHSPC [6]. Other recent additions include Lutetium-177-PSMA-617 and PARP (poly-ADP ribose polymerase) inhibitors [5]. Clinical responses to these treatments show inter-individual heterogeneity, so better tools for patient selection are required [7], [8], [9].

Prostate-specific antigen (PSA) is the gold-standard serum marker for PC and is used for both detection and monitoring purposes. Some patients with poorly differentiated tumors have low PSA despite advanced disease [10], [11]. The value of PSA is therefore limited in more advanced PC [12], [13], [14]. Thymidine kinase 1 (TK1) is a pyrimidine salvage pathway enzyme showing a cell cycle-dependent expression pattern characterized by increases during S phase followed by a decline during M phase. Tumors have higher levels of both cell proliferation and cell death, and TK1 is released into the extracellular compartment following membrane-disintegration of cycling tumor cells. The serum concentration of TK1 (sTK1) therefore reflects the proliferation rate and the extent of cell death [15]. sTK1 protein levels are associated with the Gleason score in PC [16], [17] and with tumor stage in breast cancer [18]. sTK1 enzyme activity has been used as a prognostic, predictive, and monitoring biomarker in leukemia and in Hodgkin and non-Hodgkin lymphomas [19], [20]. sTK1 enzyme levels measured via enzyme-linked immunosorbent assay (ELISA) are also elevated in breast cancer [18], [21] and PC [18], [22]. Measurement of sTK1 protein levels is superior to enzyme activity measurement with regard to sensitivity and specificity of PC detection [17], [18], [23].

A previous study investigated the value of the AroCell TK 210 ELISA for detection of PC [23]. Differences in median sTK1 protein concentrations between benign and malignant conditions were marginal. However, sTK1 increased PC detection rates when combined with PSA or the Prostate Health Index [23]. Tumor cell proliferation is more active in high-grade PC than in low-grade tumors [24], which raises the possibility that sTK1 could be a useful prognostic marker in advanced disease. Furthermore, the extent of cell proliferation in prostate tumors is expected to reflect the response to treatment with drugs that preferentially kill dividing tumor cells. The present study represents a first attempt to address the possible prognostic and treatment predictive value of sTK1 as a biomarker in metastatic PC. The study included one cohort with de novo mHSPC treated with ADT, and two cohorts with mCRPC, one treated with ARSIs and the other with docetaxel.

## 2 Patients and methods

### 2.1 Patients

This retrospective multicenter study was performed in accordance with the principles of good clinical practice and the Declaration of Helsinki.

The mHSPC cohort consisted of 43 men with synchronous hormone-naïve metastatic disease at diagnosis identified from the PC database of the Prostate Cancer Research Center of Tampere University, Finland. All men were diagnosed and treated in the urology outpatient clinic of Tampere University Hospital during 2000–2010. Metastatic disease was defined as the presence of distant metastases (stage M1) confirmed via bone imaging, regardless of T stage or N stage. Participants were randomly selected from men meeting the inclusion criteria and who had a serum sample available from the time of diagnosis. Information available included date of diagnosis, biopsy Gleason score, clinical TNM stage, PSA at the time of diagnosis, and the primary treatment method. Participants were managed according to clinicians’ discretion and standard clinical practice. Data included the time of first disease recurrence (biochemical or radiological) and the date of death from the national death certificate registry. Follow-up started from the date of diagnosis and data were available up to June 30, 2023, for a maximum of 23 yr.

mCRPC cohorts 1 and 2 included men treated at the Department of Urology, Medical University of Vienna or at the Department of Urology, Semmelweis University, Budapest between 2011 and 2022. Serum samples were collected directly before initiation of first-line systemic therapy for mCRPC; cohort 1 included 99 patients who received ARSI treatment (abiraterone or enzalutamide); mCRPC cohort 2 included 98 patients treated with docetaxel chemotherapy. Follow-up started from the date of treatment initiation and data were available up to August 2022.

### 2.2 sTK1 measurements

sTK1 was measured using the AroCell TK 210 ELISA (AroCell AB, Bromma, Sweden) according to the manufacturer’s instructions (www.arocell.com). sTK1 levels were calculated using a calibrator curve and the 4PL curve-fitting program. Each sample was analyzed in duplicate and the mean value is reported (in μg/l).

### 2.3 Statistical analysis

Analyses were carried out by Statistikakademin AB (Uppsala, Sweden). The primary endpoint was overall survival (OS). To analyze possible associations between sTK1 levels and the risk of death, the Cox proportional-hazards regression method was used to estimate hazard ratios (HRs) and 95% confidence intervals (CIs). The time metric was months since PC diagnosis for the mHSPC cohort, and months since initiation of systemic treatment for the mCRPC cohorts. Follow-up ended at death, loss to follow-up (emigration), or the cohort-specific common closing date, whichever came first.

All analyses were adjusted for age (included as a continuous variable). Multivariable adjusted analyses for the mHSPC cohort were further adjusted for biopsy Gleason score (categorized as Gleason <7, 7, and 8–10) and PSA at diagnosis (included as a continuous variable). Multivariable adjusted analyses for the mCRPC cohorts were further adjusted for PSA and Eastern Cooperative Oncology Group (ECOG) performance status (scale 0–4) [25]) at the time of CRPC treatment.

sTK1 was analyzed in a Cox regression model as a continuous variable. Furthermore, the cohorts were stratified by sTK1 quartiles to investigate OS trends with increasing TK1 level. Participants in the lowest sTK1 quartile were used as the reference group. Kaplan-Meier curves were plotted to visualize OS differences between sTK1 strata. Clinical characteristics were compared between sTK1 strata using the Mann-Whitney *U* test for continuous variables (PSA) and a χ^2^ test for categorical variables (T stage and Gleason score).

Harrell’s C index and random forest classification were used to evaluate the additional predictive value of sTK1 over established clinical prognostic factors, including Gleason grade, PSA at diagnosis, age at diagnosis, and clinical TNM stage, for prediction of PC mortality.

The study was approved by the local ethics committees of Pirkanmaa Hospital District (R03203, mHSPC cohort) and Medical University of Vienna and Semmelweis University (SE-RKEB: 33-5/2014; mCRPC cohorts). All participants gave written informed consent before collection of blood samples.

## 3 Results

### 3.1 Tumor and population characteristics

The median age at diagnosis was similar in all three cohorts (Table 1). Median PSA was 50 ng/ml in the mHSPC cohort and 96 and 68 ng/ml in mCRPC cohorts 1 and 2, respectively. In the mHSPC cohort, the majority of cases had high-grade (International Society of Urological Pathology [ISUP] grade 4–5) and locally advanced (cT3-T4) PC in addition to distant metastases. In all three cohorts, the majority of men died within the median follow-up of 17–67 mo (Table 1). The majority of men in the mCRPC cohorts had ECOG performance status of 0–1 [25].

**Table 1:** Population and clinical characteristics

Parameter	mHSPC cohort	mCRPC cohort 1 a	mCRPC cohort 2 b
Patients	43	99	98
Median age, yr (IQR) c	72 (67–76)	72 (68–78)	70 (65–74)
Median PSA at diagnosis, ng/ml (IQR)	50 (19.5–228)	96 (20.1–394)	68 (26.5–279)
ECOG performance status (*n*)			
0	NA	62	43
1	NA	19	45
2	NA	3	10
Unknown	43	15	0
ISUP grade group (*n*)			
1	6	NA	NA
2–3	15	NA	NA
4–5	32	NA	NA
T stage (*n*)			
T1	1	NA	NA
T2	7	NA	NA
T3	18	NA	NA
T4	17	NA	NA
High-volume disease (*n*) d	NA	14	13
Median FU (IQR) e			
Overall	67 (22–110)	17.2 (6.9–29.6)	22.1 (11.9–35)
For patients alive at the end of FU	130 (125–173)	36.5 (8.2–57)	34.8 (12.2–44.3)
Death (*n*)	36	72	64
Median sTK1 at baseline, μg/l (IQR)	0.61 (0.33–0.83)	0.50 (0.34–1.07)	0.49 (0.34–0.94)

sTK1 = serum thymidine kinase 1; mHSPC = metastatic hormone-sensitive prostate cancer; mCRPC = metastatic castration-resistant prostate cancer; IQR = interquartile range; ISUP = International Society of Urological Pathology; PSA = prostate-specific antigen; ECOG = Eastern Cooperative Oncology Group, NA = not available; FU = follow-up.

a Men diagnosed with CRPC and managed in the first line with either abiraterone or enzalutamide.

b Men diagnosed with CRPC and managed in the first line with docetaxel.

c Age at treatment initiation.

d According to the CHAARTED criteria [28].

e Follow-up in months of diagnosis for the mHSPC cohort or the date of treatment initiation for CRPC for mCRPC cohorts 1 and 2 to June 30, 2023 for the mHSPC cohort and August 31, 2022 for the mCRPC cohorts.

### 3.2 Serum TK1 at diagnosis

Median sTK1 at diagnosis was 0.61 μg/l (interquartile range 0.33–0.83) in the mHSPC cohort (Table 1). sTK1 levels did not markedly vary by biopsy grade (median 0.23 μg/l for grade 3–5 vs vs 0.21 μg/l for grade 1–2 tumors). Median PSA at diagnosis was higher for men with sTK1 above the median (PSA 208 ng/ml) than for men with sTK1 below the median (PSA 46 ng/ml; Supplementary Table 1). Elevated sTK1 was associated with higher clinical T stage at diagnosis (T1–2 vs T3–4; *p* = 0.021) whereas biopsy ISUP grade groups did not significantly differ by sTKI level (Supplementary Table 1).

In the mCRPC cohorts, median sTK1 was 0.50 μg/l in cohort 1 at initiation of ARSI treatment, and 0.49 μg/l in cohort 2 at initiation of docetaxel (Table 1). Using the same sTK1 cutoff (0.61 μg/l) as for the mHSPC cohort, median PSA at diagnosis in the combined mCRPC cohort was 52.2 ng/ml for men with sTK1 below the median, and 162 ng/ml for men with sTK1 above the median (*p* = 0.0006).

### 3.3 Association between sTK1 and OS in mHSPC

In the mHSPC cohort, sTK1 levels were inversely associated with shorter OS. The median survival time was 28 mo for the highest sTK1 quartile and 113 mo for the lowest sTK1 quartile (*p* for trend 0.026). The HR for association of sTK1 with OS was 7.77 (95% CI 2.25–26.9; C index = 0.82) for the highest compared to lowest sTK1 quartile after multivariable adjustment (Table 2). sTK1 as a continuous variable predicted OiS after multivariable adjustment (HR 1.47, 95% CI 1.01–2.12; C index = 0.65).

**Table 2:** Risk of overall mortality stratified by sTK1 quartiles at diagnosis in the cohort of 43 men with metastatic hormone-sensitive prostate cancer

sTK1 level	Patients	Risk of overall mortality	
		HR 1 (95% CI) a	HR 2 (95% CI) b
<0.33 μg/l	11	Reference	Reference
0.33–0.61 μg/l	11	2.66 (0.87–8.16)	3.89 (1.16–13.1)
0.61–0.82 μg/l	11	3.43 (1.24–9.49)	5.58 (1.81–17.2)
>0.82 μg/l	10	8.86 (2.92–26.9)	7.77 (2.25–26.9)

aTK1 = serum thymidine kinase 1; HR = hazard ratio; CI = confidence interval.

a Adjusted for age.

b Adjusted for age, prostate-specific antigen at diagnosis, and International Society of Urological Pathology grade group.

### 3.4 Association between sTK1 and OS in mCRPC

**Fig. 1:**
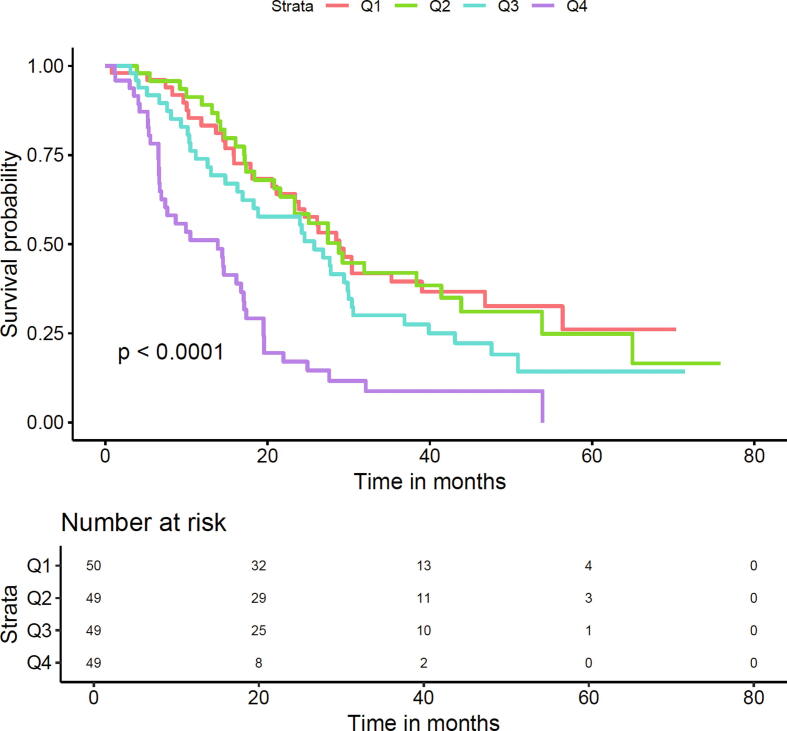
Overall survival by serum TK1 quartiles among 197 men diagnosed with metastatic castrate-resistant prostate cancer. The difference in survival among the quartiles is statistically significant (*p* < 0.001).

For the pooled mCRPC cohort, sTK1 in the highest quartile (>0.99 μg/l) was associated with higher mortality after multivariable adjustment (HR 3.10, 95% CI 1.82–5.28; Table 3 and Fig. 1).

**Table 3:** Risk of overall mortality stratified by sTK1 quartiles at diagnosis in the group of 197 men with mCRPC

sTK1 level	Risk of overall mortality											
	Pooled mCRPC cohort				mCRPC cohort 1 (enzalutamide or abiraterone)				mCRPC cohort 2 (docetaxel)			
	*n*	HR 1 a (95% CI)	HR 2 b (95% CI)	HR 3 c (95% CI)	*n*	HR 1 a (95% CI)	HR 2 b (95% CI)	HR 3 c (95% CI)	*n*	HR 1 a (95% CI)	HR 2 b (95% CI)	HR 3 c (95% CI)
<0.34 μg/l	50	Reference	Reference	Reference	25	Reference	Reference	Reference	25	Reference	Reference	Reference
0.34–0.49 μg/l	49	1.15 (0.69–1.93)	1.12 (0.66–1.88)	1.20 (0.70–2.05)	24	1.20 (0.57–2.52)	1.05 (0.49–2.26)	1.10 (0.49–2.47)	25	1.12 (0.54–2.33)	1.09 (0.52–2.29)	1.21 (0.58–2.53)
0.49–0.99 μg/l	49	1.45 (0.90–2.35)	1.46 (0.90–2.36)	1.45 (0.88–2.39)	24	2.33 (1.17–4.62)	2.13 (1.04–4.34)	1.96 (0.91–4.21)	25	0.86 (0.42–1.75)	0.85 (0.41–1.73)	1.00 (0.49–2.08)
>0.99 μg/l	49	3.63 (2.22–5.92)	3.65 (2.23–5.97)	3.10 (1.82–5.28)	26	6.36 (3.07–13.2)	6.21 (2.97–13.0)	5.22 (2.23–12.2)	23	2.38 (1.17–4.82)	2.37 (1.17–4.80)	2.28 (1.13–4.60)

sTK1 = serum thymidine kinase 1, mCRPC = metastatic castration-resistant prostate cancer; HR = hazard ratio; CI = confidence interval.

a Adjusted for age.

b Adjusted for age and prostate-specific antigen.

c Adjusted for age, prostate-specific antigen, and Eastern Cooperative Oncology Group performance status.

In mCRPC cohort 1 treated with ARSIs, sTK1 in the highest quartile was associated with higher mortality in comparison to sTK1 in the lowest quartile (age-adjusted HR 6.36, 95% CI 3.07–13.2; multivariable-adjusted HR 5.22, 95% CI 2.23–12.2; Table 3). In mCRPC cohort 2 managed with docetaxel, sTK1 in the highest quartile was also associated with significantly higher risk of death (Table 3). However, the risk was lower than that observed for mCRPC cohort 1 treated with ARSIs (age-adjusted HR 2.38, 95% CI 1.17–4.82; multivariable-adjusted HR 2.28, 95% CI 1.13–4.60).

HRs were also calculated for sTK1 as a continuous variable (Table 4). After multivariable adjustment, the association between sTK1 and shorter OS was statistically significant for the pooled mCRPC cohort and for mCRPC cohort 1 treated with ARSIs, but not for mCRPC cohort 2 treated with docetaxel.

**Table 4:** Association of overall mortality with sTK1 as a continuous variable in the group of 197 men with mCRPC

	Pooled mCRPC cohort (*n* = 197)	mCRPC cohort 1 (Enza/Abi; *n* = 99)	mCRPC cohort 2 (docetaxel; *n* = 98)
HR 1 (95% CI) a	1.09 (1.06–1.13)	1.08 (1.05–1.12)	1.09 (1.01–1.17)
HR 2 (95% CI) b	1.09 (1.06–1.13)	1.08 (1.04–1.12)	1.09 (1.02–1.17)
HR 2 (95% CI) c	1.08 (1.04–1.22)	1.08 (1.03–1.13)	1.07 (0.99–1.15)

sTK1 = serum thymidine kinase 1; mCRPC = metastatic castration-resistant prostate cancer; Enza = enzalutamide; Abi = abiraterone; HR = hazard ratio; CI = confidence interval.

a Adjusted for age.

b Adjusted for age and prostate-specific antigen.

c Adjusted for age, prostate-specific antigen, and Eastern Cooperative Oncology Group performance status.

### 3.5 Added prognostic value of sTK1 in mHSPC

The random forest classification accuracy for OS was highest when using PSA, age, TK1 and ISUP Gleason grade group as classifiers (Supplementary Fig. 1). This model including TK1 had higher prediction accuracy for mean OS than the use of established prognostic clinical characteristics alone.

## 4 Discussion

We demonstrated that serum TK1 could be used as a prognostic factor in metastatic PC in both in the hormone-sensitive and castration-resistant settings, especially for patients treated with ADT and ARSIs. The marker remained prognostic after adjustment for established clinical risk factors.

The mHSPC cohort consisted of men diagnosed with de novo metastatic disease during 2000–2010. Stratification of this group by median sTK1 clearly divided patients into subgroups with sharply differing prognosis after adjustment for age, PSA at diagnosis, and tumor Gleason score. This suggests that sTK1 could be used to select men for intensive treatment schemes, such as triplet therapy. However, men in our mHSPC cohort were managed according to the treatment standard at the time, which was ADT monotherapy. Furthermore, detection of metastases at the time was based on CT and bone scan as prostate-specific membrane antigen (PSMA) positron emission tomography was not yet available. Therefore, our results need to be validated in contemporary cohorts that have been managed using current diagnostics and standards of care.

We found that sTK1 levels also showed prognostic value in mCRPC. The risk association was stronger for patients treated with ARSIs than for patients treated with docetaxel (age-adjusted HRs of 6.36 and 2.38 for the upper and lower quartiles, respectively). This observation raises an intriguing question regarding whether sTK1 could also be used as a marker for predicting if a patient might benefit from docetaxel rather than ARSI therapy. This issue requires further validation in a prospective cohort setting in which patients are randomized to different lines of standard treatments. Our findings are consistent with the literature and with underlying biology considering that high sTK1 levels are associated with aggressive disease [17] and that the effect of ADT is limited against Gleason 9–10 PC [26]. Therefore, sTK1 levels could reflect tumor aggressiveness and could thus predict the response to androgen receptor–targeted therapies. However, this hypothesis needs to be verified in a prospective analysis or using blood samples from trials that specifically tested for the effects of ARSIs or docetaxel in a randomized setting (eg, CHAARTED and STAMPEDE) [27], [28].

Rapidly proliferating PC cells are expected to be sensitive to drugs that impact the cell cycle, such as docetaxel, which is an inhibitor of microtubule dynamics and mitosis. Transcriptomic analysis has revealed that docetaxel-sensitive breast tumors had higher expression of genes involved in the cell cycle [29]. Thymidine kinase is a downstream target in the Rb/E2F pathway [30]. Screening for cancer drugs effective against cancers with defects in the Rb pathway resulted in identification of mitotic inhibitors, supporting the notion that patients with high sTK1 levels benefit more from docetaxel chemotherapy [31].

In contrast to PC, a study in patients with breast cancer demonstrated similar performance for sTK1 enzyme activity and sTK1 protein levels [18]. Recent studies suggest that sTK1 activity could be a potentially useful biomarker for therapeutic decision-making in breast cancer. Elevated sTK1 activity levels before treatment with cyclin-dependent kinase 4/6 inhibitors and subsequent weak suppression of sTK1 activity during treatment were associated with poor prognosis for patients with hormone receptor–positive/HER2-negative metastatic breast cancer [32]. Furthermore, a randomized trial involving patients with metastatic breast cancer who received different types of endocrine therapy demonstrated that sTK1 activity was a treatment predictive biomarker [33].

A strength of our study is that it is the first exploration of the prognostic value of sTK1 in metastatic PC. Furthermore, we evaluated sTK1 in both the mHSPC and mCRPC settings, with adequate follow-up to estimate OS as an endpoint.

Our study has several limitations. We did not have information on specific causes of deaths in the mCRPC cohorts and thus were not able to conduct a competing-risks analysis. Therefore, our results could reflect the overall poorer health of men with high sTK1. Another limitation is that the mCRPC cohort was not randomized for ARSIs or docetaxel treatment, leading to potential selection bias. Nevertheless, the distributions of both PSA and sTK1 levels were similar between these subgroups and the differences in HRs remained after statistical adjustments. We also lacked information on metastatic volume for the mHSPC cohort and on prior treatments for PC in the mCRPC cohorts. Randomized prospective studies are needed to validate the predictive value of sTK1 in different populations and clinical scenarios.

## 5 Conclusions

sTK1 is a promising biomarker for risk stratification of patients with mHSPC or mCRPC. The association between elevated sTK1 and poor OS was particularly strong for men treated with androgen-targeted therapies. Even though our results are promising, confirmation is needed from randomized studies of treatments for advanced PC. Demonstration that sTK1 can predict the efficacy of a randomized treatment intervention would confirm its value as a predictive biomarker.

***Author contributions****:* Teemu J. Murtola had full access to all the data in the study and takes responsibility for the integrity of the data and the accuracy of the data analysis.

*Study concept and design:* Murtola, Linder, Kramer, Szarvas.

*Acquisition of data:* Murtola, Tammela, Kramer, Szarvas, Siltari.

*Analysis and interpretation of data:* Murtola, Linder, Kramer, Szarvas, Siltari, Raittinen.

*Drafting of the manuscript*: Murtola, Siltari, Linder, Kramer, Szarvas.

*Critical revision of the manuscript for important intellectual content:* Murtola, Siltari, Raittinen, Tammela, Linder, Csizmarik, Kramer, Szarvas.

*Statistical analysis:* Berglund, Statistikakademin AB Uppsala, Siltari, Raittinen.

*Obtaining funding:* Murtola.

*Administrative, technical, or material support:* None.

*Supervision:* None.

*Other*: None.

***Financial disclosures****:* Teemu J. Murtola certifies that all conflicts of interest, including specific financial interests and relationships and affiliations relevant to the subject matter or materials discussed in the manuscript (eg, employment/affiliation, grants or funding, consultancies, honoraria, stock ownership or options, expert testimony, royalties, or patents filed, received, or pending), are the following: Teemu J. Murtola reports lecture fees from Astellas, Janssen, Novartis, and Sanofi, and a paid consultant role for Astellas and Janssen. Teuvo L.J. Tammela reports a paid consultant role for Astellas, GSK, Pfizer, Orion Pharma, and Amgen. Stig Linder reports a paid consultant role for AroCell AB. The remaining authors have nothing to disclose.

***Funding/Support and role of sponsor****:* This study received material support from AroCell AB in the form of TK 210 ELISA kits. The sponsor was not involved in interpretation of the data but had a role in preparation, review, and approval of the manuscript. The study was also funded by grants from the Academy of Finland (#330724), the Nordic Cancer Union (#R280-A16023), and Cancer Foundation Finland (#210054).

***Acknowledgements****:* We thank Maria Hägg Olofsson and Paola Pellegrini for technical assistance, and Anders Berglund (Statistikakademin, Uppsala) for statistical evaluations.
