# Supplementary figures and images for: Elevated Levels of Serum Thymidine Kinase 1 Predict Poor Survival for Patients with Metastatic Prostate Cancer
Source: Eur Urol Open Sci. 2024 Oct 25;70:135–41. doi: 10.1016/j.euros.2024.10.010 (PMC11547961; doi:10.1016/j.euros.2024.10.010)

## Random forest classification error using various set of features

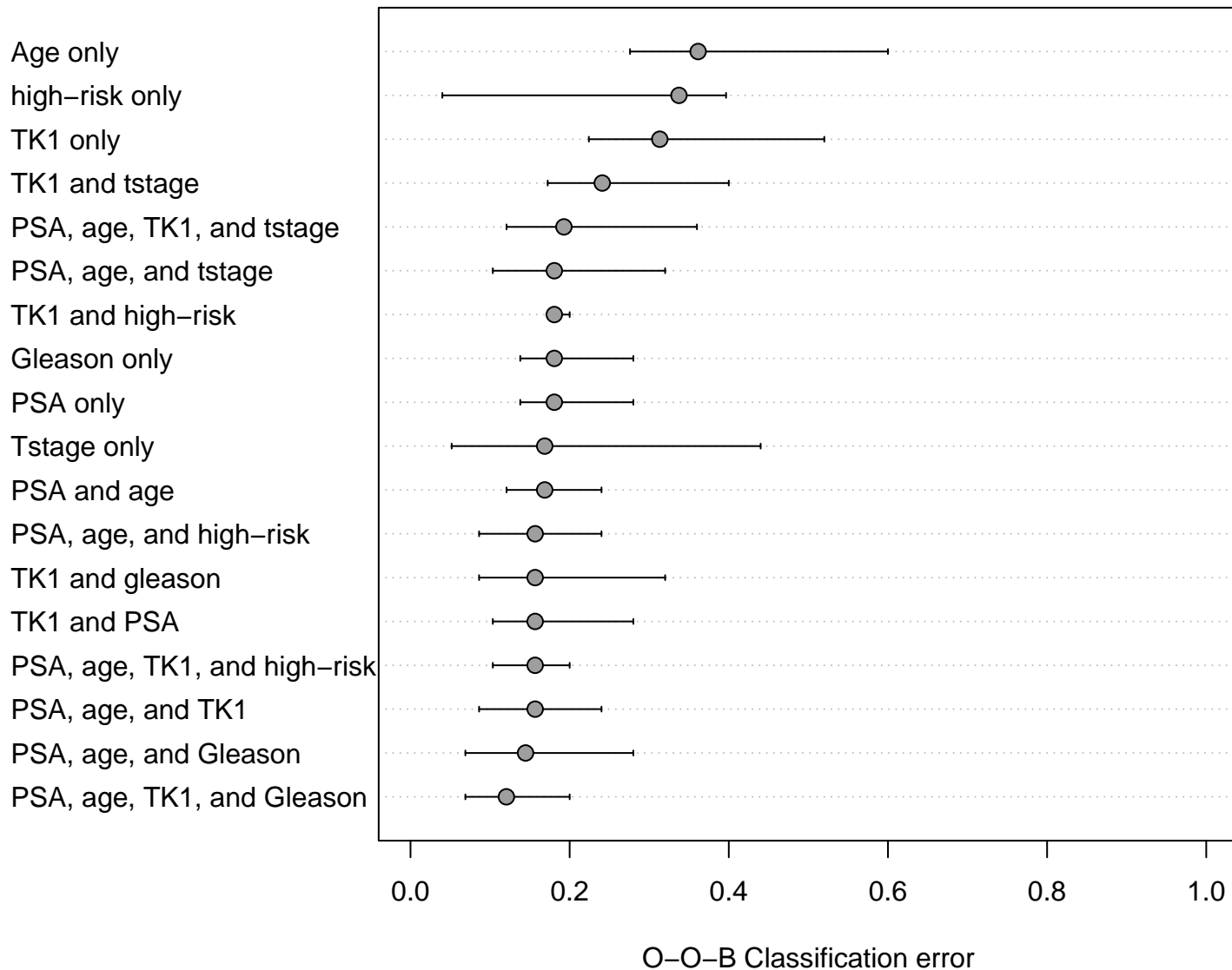

Supplement: Supplementary Data 1 [file mmc1.pdf]
