# Supplementary material for: Elevated Levels of Serum Thymidine Kinase 1 Predict Poor Survival for Patients with Metastatic Prostate Cancer
Source: Eur Urol Open Sci. 2024 Oct 25;70:135–41. doi: 10.1016/j.euros.2024.10.010 (PMC11547961; doi:10.1016/j.euros.2024.10.010)
Supplement: Supplementary Data 2 [file mmc2.docx]

**Supplementary Table 1.** Clinical characteristics in mHSPC cohort by median serum thymidine kinase 1 (sTK1) level (above or below 0.61).

|  | sTK1 levels at diagnosis (μg/L) | | p-value |
| --- | --- | --- | --- |
|  | < 0.61 | > 0.61 |  |
| PSA (median, IQR)  (ng/mL) | 46 (17; 70) | 208 (27; 828) | 0.013 |
| ISUP Gleason (n)  1-2  3-5 | 6  16 | 5  16 | 0.8 |
| T-stage (n)  T1-T2  T3-T4 | 1  21 | 7  14 | 0.021 |

**Supplementary Figure 1.**

Random forest classification error for prediction of overall survival of mHSPC patients in models including sTK1 in combination with clinical prognostic factors ISUP Gleason grade group, clinical T-stage, PSA and age at diagnosis.

Predictive values of the set of prognostic factors were ranked by the classification error per model, where lower classification error indicated better model prediction. Each random forest model was repeated 1000 times for empirical confidence interval estimation using percentile method for the obtained 1000 error estimates, and the median of these as the point estimate for the classification error.
